# Supplementary material for: A computational text analysis of recent African digital health strategies and their attention to vulnerable populations
Source: PLoS One. 2026 May 19;21(5):e0348593. doi: 10.1371/journal.pone.0348593 (PMC13186356; doi:10.1371/journal.pone.0348593)
Supplement: S1 File — (DOCX) [file pone.0348593.s001.docx]

**Annex**

**Table A1. Topic Labelling.**

| **Key Terms/Word Stems** | **Assigned Label** | **Exemplary Strategy Excerpt** |
| --- | --- | --- |
| management, resource, level, plan, quality, sector, implementation, care, facility, application, decision, tool, access, infrastructure, development, availability, research, capacity, data, disease | 1: Digital Health Capacity Building | *„Successful implementation of the eHealth strategy requires a pool of skilled and competent manpower at all implementation levels. It is therefore critical that a comprehensive eHealth human resource development strategy be developed and implemented to address manpower shortages and technical capacity at central, district, hospital and facility levels.” [Namibia]* |
| solution, implementation, facility, management, initiative, level, sector, delivery, resource, governance, activity, quality, standard, community, government, care, development, infrastructure, requirement, guideline | 2: Health System Reform and Transformation | *„In order to reform healthcare governance, improve its quality and efficiency, and reduce still significant health inequalities, this far-reaching reform will involve (i) a continued upgrade and expansion of the healthcare offer in a fair and equitable manner; (ii) a reduction of the significant deficit in human resources, strengthening their capacities and enhancing the value of public health service; (iii) a consecration of the regional dimension in the sector’ management by positioning university hospitals as real driving engines of the region; (iv) the convergence of public policies on disease prevention and control; (v) consecrating the private sector as a real partner in this reform process; and (vi) the digital transformation of the sector through the creation of an integrated national health information system (NHIS)*.” [Morocco]* |
| care, intervention, sector, level, facility, child, quality, disease, development, year, population, community, management, medicine, hospital, program, access, government, country, implementation | 3: Digital Health for Targeted Care | *„All levels of the health pyramid — from national tertiary hospitals down to the community level — are concerned. Digital health should facilitate true continuity of care with holistic, longitudinal patient follow‑up. … The systematic use of treatment protocols for malaria, TB, HIV, and hepatitis should be enabled through integrated clinical decision support tools within the electronic patient record. Vaccination coverage management tools should also be integrated.” [DR Congo]* |
| implementation, solution, sector, development, level, blueprint, stakeholder, standard, access, intervention, infrastructure, workforce, policy, research, decision, initiative, delivery, area, quality | 4: Stakeholder-driven Digital Health Design and Implementation | *“Finally, it is recommended that any IS project should integrate the various stakeholders from as early as the design phase, thus facilitating its adoption and minimizing the risks associated with interoperability.” [Morocco]* |
| implementation, plan, worker, management, user, development,  patient, research, framework, ict, standard, policy, training, solution,  application, stakeholder, directorate, intervention, investment,  facility | 5: Interoperability and Organizational Alignment | *“In addition, the Zanzibar Health Policy 2011 clearly articulates lack of an integrated health information system (HIS) to enable information sharing from various sub-systems; and thus, recommending development and implementation of a comprehensive ICT strategy across the health sector to support such functions as general office management, data sharing among health systems, telehealth, and e-learning.” [ Zanzibar]* |
| policy, level, resource, management, implementation, ict, training, plan, infrastructure, quality, number, intervention, division, staff, care, district, disease, committee, unit, delivery | 6: Digital Health Governance and Policy | *“The situation analysis has covered issues of leadership, governance and Legislation, Policy and Compliance [..] Furthermore,*  *lack of a minimum set of standards with criteria against which EHRs can be measured has made difficult the coordination effort to harmonize effort around EHR*  *implementation. These challenges point to inadequate policy and regulatory Framework for the management and coordination of digital health implementations.” [Malawi]* |
| development, care, plan, objective, level, implementation, state, activity, communication, infrastructure, population, intervention, facility, management, application, coverage, country, act, area | 7: Strategic Management of Digital Health Interventions | *„Inevitably, the implementation of this strategy is not without risks. When risks overshadow the ability to succeed the attainment of objectives can be constrained. To avoid failure in the implementation it is important to take a strategic approach to anticipate any potential obstacles for implementation and constitute mitigation strategies.” [Malawi]* |
| patient, professional, user, care, solution, development, sector, application, term, digitisation, country, telemedicine, hospital, player, security, transformation, exchange, implementation, project | 8: User-centered Digital Health | *“Systems emerging from the strategy should be designed with the user in mind; and the use of the system should ensure a better experience of the health system, thereby providing more patient-centric, health worker-centric and citizen-centric services.” [Namibia]* |
| development, government, sector, area,  infrastructure, ict, country, year,  coordination, objective, policy, skill,  education, framework, access,  cybersecurity, implementation,  business, regulation | 9: Coordinated and Integrated Digital Health Implementation | *“Several emerging digital health*  *interventions can help address the challenges of the health system at different levels, throughout the process leading to universal health coverage." [Cameroon]* |

**Table A2. Topic Labelling Iterations.**

| **#** | **1^st^ Iteration Topic Label** | **2^nd^ Iteration Topic Label** | **Reason for Change** | **3^rd^ Iteration Topic Label** | **Reason for Change** |
| --- | --- | --- | --- | --- | --- |
| 1 | Digital Health Capacity Building and System Readiness | Digital Health Capacity Building | “System” not inherent in Key Terms | / | / |
| 2 | Integrated Healthcare Development and Infrastructure Reform | Health System Reform and Transformation | Not only infrastructure is being reformed | / | / |
| 3 | Digital Health for Targeted Care Delivery | Digital Health for Targeted Care | “Care” already implies “Delivery” | / | / |
| 4 | Systemic Implementation of Digital Health | Stakeholder-driven Digital Health Implementation | “Systemic” not at the core but the people who implement it | Stakeholder-driven Digital Health Design and Implementation | People who design it should be the focus and implementation should be a clear separate step |
| 5 | Organizational Alignment and Coordinated Digital Health Deployment | Organizational and Strategic Alignment in Digital Health Implementation | Add strategic dimension; reframe deployment as implementation | Interoperability and Organizational Alignment | Refocus on interoperability dependency; drop generic implementation |
| 6 | Governance and Policy Frameworks for Digital Health | Digital Health Governance and Policy | Front-load domain; drop redundant “framework” | / | / |
| 7 | Strategic Planning and Risk Management in Digital Health | Strategic Risk Management in Digital Health | Drop redundant “planning” | Strategic Management of Digital Health Interventions | “Risk Management” implied in “Strategic Management” |
| 8 | Patient-centered Digital Health Implementation | Patient-centered Digital Health | Focus is not necessarily on implementation | User-centered Digital Health | Users are not necessarily patients |
| 9 | Ecosystem-approach to Digital Health | Coordinated Digital Health Implementation | Shift from conceptual lens to implementation focus | Coordinated and Integrated Digital Health Implementation | Highlight cross-system integration beyond mere coordination |
