# Supplementary material for: A computational text analysis of recent African digital health strategies and their attention to vulnerable populations
Source: PLoS One. 2026 May 19;21(5):e0348593. doi: 10.1371/journal.pone.0348593 (PMC13186356; doi:10.1371/journal.pone.0348593)
Supplement: Attachment — Submitted filename: Reviewer feedback.docx [file pone.0348593.s002.docx]

This manuscript addresses a highly relevant and timely issue: whether African national digital health strategies meaningfully address vulnerable populations at risk of digital exclusion. The study is well-positioned, theoretically grounded, and methodologically transparent. The combination of topic modelling (LDA) and dictionary-based analysis is appropriate for the research question and scale of data.

However, while the paper is methodologically sound and clearly written, it would benefit from:

Kindly check a few areas below that need improvement:

1. reducing lengthy and dense sentences (e.g., lines 389–392: “However, solely focusing on accessibility of digital health interventions overlooks other critical digital divide issues…”) and could be broken down for readability.

2. Avoid repetitive points, e.g., concepts like “vulnerable groups are acknowledged but not operationalized” and “risk of reinforcing inequities” are repeated multiple times. While emphasis is important, too much repetition can dilute the discussion. Suggest condensing to highlight distinct implications or mechanisms.

3. Provide concrete examples of strategy documents that illustrate the identified gaps (e.g., lack of age-specific considerations, superficial focus on women and children). You may include brief examples or quotes from policies that would make the discussion section more persuasive.

4. The discussion points out gaps but doesn’t clearly suggest next steps or frameworks that could improve equity. For example, after highlighting that elderly populations are neglected, the discussion could briefly propose including age-targeted digital literacy programs or user-centered co-design approaches

5. Improve terminology clarity, such that some phrases could be more precise. For example, “algorithmic awareness” may not be universally understood; consider defining or briefly explaining it. “Digitizing existing health services” could be expanded to clarify what this entails—e.g., telemedicine platforms, mobile apps, and electronic health records.
