# Supplementary material for: A computational text analysis of recent African digital health strategies and their attention to vulnerable populations
Source: PLoS One. 2026 May 19;21(5):e0348593. doi: 10.1371/journal.pone.0348593 (PMC13186356; doi:10.1371/journal.pone.0348593)
Supplement: Attachment — Submitted filename: Reviewer_Feedback_Digital_Health_Strategies.docx [file pone.0348593.s003.docx]

# Feedback

Thank you for the opportunity to review this manuscript. I found this to be a thoughtful, timely, and well-executed study that addresses an important gap in how national digital health strategies engage with equity and vulnerable populations in African contexts. The paper is clearly written, methodologically transparent, and grounded in a strong body of literature. Overall, it makes a valuable contribution to conversations on digital health policy and the digital divide.

That said, I have a few suggestions that I believe would strengthen the manuscript further and improve clarity, nuance, and policy relevance.

Major Comments

**1. Framing of “vulnerable populations.”**
The focus on women, children/youth, and older adults is well motivated and aligns with much of the existing literature on digital exclusion. However, vulnerability is highly context-specific and often intersectional, particularly in African settings. Readers may wonder why other commonly marginalized groups—such as people with disabilities, rural or remote populations, ethnic minorities, or refugees—are not included.
While this is acknowledged briefly in the limitations, clarifying the rationale for selecting these three groups earlier in the paper would help frame the scope of the analysis more clearly.

**2. Interpretation of topic modeling results**
The topic modeling analysis is carefully conducted and clearly explained. However, the absence of vulnerable populations as distinct topics does not necessarily imply policy neglect; rather, it suggests they are not salient enough in the text to emerge as standalone themes. These populations may still be embedded within broader topics such as service delivery or user-centered design. A brief reflection on the limits of topic modeling would strengthen the interpretation.

**3. Dictionary-based analysis and robustness**
The dictionary approach is appropriate and adds depth to the analysis. Readers may appreciate additional detail on how the dictionaries were developed and validated, how ambiguous terms were handled, and how translation of non-English documents may have affected results.

**4. Strengthening the policy implications**
The discussion compellingly argues that superficial references to vulnerable groups risk reinforcing inequalities. Including concrete examples of what “equity-by-design” could look like in digital health strategies (e.g., explicit equity targets, digital literacy initiatives, accessibility standards) would enhance policy relevance.

**5. Scope and generalizability**
While the inclusion of 13 national strategies is reasonable, the discussion could more explicitly acknowledge regional diversity across Africa and clarify that findings reflect available and recent strategies rather than all national approaches.

**Minor Comments**
- A light proofreading pass would address minor typographical and formatting issues.
- Some long sentences could be shortened for readability.
- Table 3 is informative but dense; simplifying it or moving some content to the appendix may improve clarity.

Overall Recommendation: Major Revision

This is a strong and important manuscript. The suggested revisions focus on clarifying scope, strengthening methodological nuance, and enhancing policy relevance. Addressing these points would substantially strengthen the paper.
