# Supplementary material for: A computational text analysis of recent African digital health strategies and their attention to vulnerable populations
Source: PLoS One. 2026 May 19;21(5):e0348593. doi: 10.1371/journal.pone.0348593 (PMC13186356; doi:10.1371/journal.pone.0348593)
Supplement: Attachment — Submitted filename: Response to Reviewers.docx [file pone.0348593.s005.docx]

Dear Dr. Raimi, dear editorial team, dear reviewers,

Thank you for your valuable comments on our manuscript and to the Academic Editor for facilitating the revision process.

We have made the necessary changes to our manuscript, and believe that your feedback helped improve our submission immensely. Please find our point-by-point responses to the reviewer comments and the required changes according to the decision letter in the response tables below.

Sincerely,

Kimsey Zajac, Louisa Peters, Lutz Kolbe

| **Comments from Reviewer #1** | **Authors’ Response** |
| --- | --- |
| 1. reducing lengthy and dense sentences (e.g., lines 389–392: “However, solely focusing on accessibility of digital health interventions overlooks other critical digital divide issues…”) and could be broken down for readability. | Thank you for your comment. We tried to be mindful of this in the revision and hope to have improved reading flow. Besides trying to make sentences clearer, we have also adapted the formatting of the results section, so that the quotes are now separated from the main text, which will also improve readability. |
| 2. Avoid repetitive points, e.g., concepts like “vulnerable groups are acknowledged but not operationalized” and “risk of reinforcing inequities” are repeated multiple times. While emphasis is important, too much repetition can dilute the discussion. Suggest condensing to highlight distinct implications or mechanisms. | Thank you for this suggestion. We agree that these repetitions can be avoided. We have hence condensed the relevant sections by removing redundant statements and merging overlapping passages, particularly in the Discussion. |
| 3. Provide concrete examples of strategy documents that illustrate the identified gaps (e.g., lack of age-specific considerations, superficial focus on women and children). You may include brief examples or quotes from policies that would make the discussion section more persuasive. | Thank you for your comment. We have deliberately refrained from including additional quotes in the discussion section, as the results section already contains numerous illustrative examples, including those directly related to the point you raise (see Chapter “Inclusivity of National Digital Health Strategies”). To improve clarity, we have slightly revised the formatting of the results section to make the existing quotes more prominent. We are happy to include even more quotes, if desired, however, they will be very similar to the ones already included. |
| 4. The discussion points out gaps but doesn’t clearly suggest next steps or frameworks that could improve equity. For example, after highlighting that elderly populations are neglected, the discussion could briefly propose including age-targeted digital literacy programs or user-centered co-design approaches. | Thank you for pointing this out. We have included some recommendations in the discussion and conclusion. |
| 5. Improve terminology clarity, such that some phrases could be more precise. For example, “algorithmic awareness” may not be universally understood; consider defining or briefly explaining it. “Digitizing existing health services” could be expanded to clarify what this entails—e.g., telemedicine platforms, mobile apps, and electronic health records. | Thank you for this suggestion. Some terms could indeed be clearer for a broad readership. We therefore added brief in-text definitions for algorithmic awareness and offered concrete examples for “digitizing existing health services”. |

| **Comments from Reviewer #2** | **Authors’ Response** |
| --- | --- |
| Relevance: This topic has huge relevance, particularly in the context of growing consideration for digital health within low as well as middle-income countries. It has been noted that the attention paid to vulnerable groups in this topic removes a very crucial lacuna contained within existing knowledge.  Methodology: The application of computational text analysis, especially using Latent Dirichlet Allocation, is a strong methodology for theme priority analysis in a set of several documents. This methodology framework increases authenticity in results.  Clear Objectives: The objectives of this study are well-articulated and include examining the inclusiveness of national digital health strategies. The study's emphasis on gender and age disparities is quite praiseworthy.  Holistic Data Set: Using 13 digital health strategies from across Africa can make this research more robust. The use of varied data can present its findings from different perspectives. | // |
| Level of Analysis: The analysis has a level of statistics but may require additional levels of analysis. Quotes or examples from the strategies may add further demonstration of the level in which vulnerable groups have been considered. | Thank you for your comment. Quotes from the strategies have been included, but we have adjusted the formatting to make this clearer. The main finding from this analysis was that the inclusion is only superficial; we have tried to make this a bit clearer in the discussion. |
| Discussion of Findings: The findings section of the essay might expand on the implications of the findings. The discussion of the gaps that have been identified can help support the case for the change of policy based on the implications on health inequalities. | We have tried to expand a bit on this part in the discussion; however, we also want to be mindful of repetition (as another reviewer commented). We hope that the current version of our discussion is an improvement. |
| Policy Recommendations: While the recommendations suggested in the conclusion-to-be are to incorporate equity targets, more detailed yet actionable directives might help make this paper more effective for policymakers. | Thank you for pointing this out. We have included actionable guidance in the conclusion. |
| Other Populations to be Considered: It is pertinent to note that highlighting the role of women, children, and the aged is important, but there could be mention of other sections of the population, such as the disabled or the minorities, to offer a balanced perspective. Section on Limitations  Actually, an explanation of the limitations associated with this study and their generalizability implications could be valuable. For example, it is probable that the findings are affected by the existence of some language barriers. | Thank you. This point was also raised by the other reviewers and we agree that we need to explain this more. We have tried to expand the limitation section in this regard. |

| **Comments from Reviewer #3** | **Authors’ Response** |
| --- | --- |
| This is a well-written and presented manuscript. The introduction provides a strong foundation, clearly outlining the concept of digital health and its relevance to advancing universal health coverage. It also defines “vulnerable populations” within the scope of the study. While I have some reservations about how vulnerability is conceptualized in the context of digital health in Africa and about the specific groups identified by the authors as vulnerable, the manuscript nevertheless offers a reasonable justification for how these populations are structurally marginalized within the African context. I will expound on these concerns later in the review. The manuscript offers a timely and highly relevant computational text analysis of 13 national digital health strategies from African countries, all published since 2020. Using Latent Dirichlet Allocation to identify thematic priorities and a dictionary-based approach to quantify references to women, children, and older adults, the study indicates that these strategies largely prioritize systemic and infrastructural development, while the inclusion and equity needs of vulnerable populations are often addressed superficially or overlooked. This central claim is articulated and represents an important contribution, as it provides quantitative evidence that current policy approaches may inadvertently widen the digital divide, a longstanding concern in global and digital health. The study is significant because it offers a systematic and quantitative assessment of digital health policy documents in a critical region. The findings are well situated within existing literature on the digital divide, digital health in low- and middle-income countries, and the WHO’s agenda for inclusive digital transformation. The authors engage with the literature appropriately, and the data and analyses largely align with and support their conclusions. Overall, the methodology is sound and well aligned with the research objectives, and it is presented in a manner that remains accessible to non-specialist readers while retaining sufficient technical rigor. | // |
| There are, however, a few areas where revisions would strengthen the manuscript. Lines 94–100 would be better placed in the Methods section, as they describe the document analysis approach and largely repeat material already presented there. In addition, while the authors report using Python and standard natural language processing libraries, transparency and reproducibility would be improved if the scripts and code used for the analysis were made openly available in a public repository.  Overall, the manuscript demonstrates clear potential and would benefit from revision rather than rejection. | Thank you for your valuable comments (most are addressed in more detail below). Regarding the comment on Lines 94-100: We have moved the respective section to the methods section. |
| Data selection and justification (N = 13, post-2020 cut-off)  The authors’ decision to restrict the analysis to national digital health strategies published from 2020 onwards (Line 116) is an important methodological choice and needs stronger justification. While the authors link this cut-off to the WHO Global Strategy on Digital Health 2020–2025 as shown in lines 118–120, the result is a relatively small sample of 13 strategies. This raises questions about the generalizability of the findings across the African continent (13 out of 54).  I would encourage the authors to engage more explicitly with the implications of excluding pre-2020 strategies, particularly if earlier documents may have reflected different policy priorities. The rationale for why a sample of 13 strategies is sufficient to make continent-wide claims should also be strengthened. In addition, a sensitivity analysis or a more detailed discussion of how the inclusion of older strategies might affect the topic modeling results would help journal readers better judge the robustness of the findings. | Thank you – you raise an important point with this comment. The time cut-off was a strategic choice due to the fact that we wanted to analyze digital health strategies that were still up-to date by the time of data collection. Further, limiting the analysis to strategies published during this period increases conceptual comparability across countries and ensures that the identified priorities are situated within a shared global policy framework, since the publication of the WHO Global Strategy on Digital Health 2020–2025 marked a significant shift toward more coordinated, system-level approaches to digital health. We have tried to make both points clearer, but have also highlighted the point regarding generalizability in the limitations and point to possible future research avenues that could look at the inclusivity of digital health strategies over time (e.g., how and whether inclusivity has evolved over time). We have also adapted our title and introduction to reflect the focus on recent strategies. |
| Scope of “vulnerable populations”  As I mentioned above, I have a reservation concerning the authors’ definition of “vulnerable population.” The focus on women, children, and older adults (Table 2, Line 203) as the sole representations of structurally marginalized populations is quite narrow for an equity-focused analysis. While these groups are undeniably important, vulnerability in the context of digital health in Africa extends beyond age and gender. It also includes people with disabilities, rural and remote populations, individuals with low literacy or digital literacy, and, in many contexts, ethnic or linguistic minorities.  This limitation should be acknowledged more clearly in the discussion. The authors may also wish to reflect on whether their dictionary-based approach could reasonably be expanded to include additional dimensions of vulnerability, such as disability, rurality, literacy, or access, even if only in an exploratory way. | This is an equally important point that has also been raised by the other reviewers. We tried to make this point clearer in the paper, highlight it more the limitations but also explain our reasoning for focusing on these three groups a bit better. |
| Reproducibility and data availability  If they haven’t already, the authors should make available in a public repository the preprocessed text corpus used for LDA modeling, the complete dictionary of terms used in the analysis, and the code used to run the models, including the parameters that produced the final nine-topic solution. This is particularly important given PLOS ONE’s emphasis on reproducibility for computational research. | Thank you for your comment. We are happy to provide our python script and data. We have included a link to online supplementary material in the method section (<https://doi.org/10.6084/m9.figshare.31169590>). |
| Minor comments  Table 3: Topical Priorities of National Digital Health Strategies (Line 205)  In the four-column table, the first column has no heading. In addition, in Topic 4, the description in the first column (“Enabling Conditions for Digita”) is incomplete, as the text is cut off and not fully visible within the cell. | The formatting issues are resolved due to splitting the table based on suggestions by another reviewer. |
| Additional data source clarification  The study methodology does not mention the use of interviews or any other qualitative data collection methods. However, part of the results presents statements attributed to personnel; for example, lines 233–234 read, “…many doctors and nurses believe that meeting ICT requirements is a burden that takes them away from their primary tasks (Zambia).”  Could the authors please clarify the source of such information?  Furthermore, provide the source of this information in lines 237–240. (“In the most recent training needs assessment, 40% of facilities did not have staff who formally provided IT/systems support. At facilities that did, they lacked the training and mastery levels in key areas of competency for such a role.” | The quotes included stem directly from the digital health strategies. We have included a sentence at the beginning of the chapter that hopefully makes this more clear. |
| Clarity in results presentation  In the Results section, the authors note that women and children are often mentioned in relation to specific health programs, such as maternal and child health. Since the dictionary-based analysis only captures term frequency, including a small number of illustrative quotes from the strategies would help demonstrate the context of these mentions and strengthen the argument that they are largely superficial. | There are some quotes from the strategies included; we have tried to make this a bit more obvious by explicitly mentioning this at the beginning of the chapter and by adjusting the formatting. |
| Language and terminology  The manuscript is generally clear and well written, but there is some inconsistency in terminology. The title refers to “vulnerable populations,” the abstract mentions “vulnerable and marginalized groups,” and the introduction uses “structurally marginalized populations.” While these terms are closely related, briefly clarifying the preferred umbrella term and how it is defined would improve clarity. | Thank you for pointing this out; we now consistently used the term “vulnerable populations”. |
| Repeated word  Line 291, repeated word “strategic management of digital health interventions includes recognizing potential potential” | Removed |
| References  While all the references checked out, the authors should consider adding web links (URLs or DOIs) to all references to improve accessibility and enhance transparency. | We have adapted the citation style so that the web links should be accessible now. |
| Final recommendation  Overall, this study addresses an important gap in the literature using a sound methodological approach and generates findings that are both timely and relevant. The manuscript is clearly written, and the conclusions are largely supported by the analysis.  I observed two main methodological issues. First, the generalizability of the findings is limited, as only 13 out of the 54 African countries were appraised. Second, the definition of vulnerable populations in digital health contexts appears narrow. Beyond women, children, and the elderly, vulnerable groups also include people in rural and hard-to-reach settings, individuals with low literacy levels, and ethnic minorities within countries.  I would recommend that the authors be encouraged to resubmit a revised version, with particular attention to strengthening the justification for the sample selection and ensuring full transparency and reproducibility of the data and code. | // |

| **Comments from Reviewer #4** | **Authors’ Response** |
| --- | --- |
| 1. Framing of “vulnerable populations.”  The focus on women, children/youth, and older adults is well motivated and aligns with much of the existing literature on digital exclusion. However, vulnerability is highly context-specific and often intersectional, particularly in African settings. Readers may wonder why other commonly marginalized groups—such as people with disabilities, rural or remote populations, ethnic minorities, or refugees—are not included.  While this is acknowledged briefly in the limitations, clarifying the rationale for selecting these three groups earlier in the paper would help frame the scope of the analysis more clearly. | Thank you for this comment. We have now also included this in the introduction and hope that the rationale for choosing these three groups now becomes clearer. |
| 2. Interpretation of topic modeling results  The topic modeling analysis is carefully conducted and clearly explained. However, the absence of vulnerable populations as distinct topics does not necessarily imply policy neglect; rather, it suggests they are not salient enough in the text to emerge as standalone themes. These populations may still be embedded within broader topics such as service delivery or user-centered design. A brief reflection on the limits of topic modeling would strengthen the interpretation. | Thank you for this comment; indeed, this did not become clear in our wording. We have included a sentence reflecting this in the discussion. |
| 3. Dictionary-based analysis and robustness  The dictionary approach is appropriate and adds depth to the analysis. Readers may appreciate additional detail on how the dictionaries were developed and validated, how ambiguous terms were handled, and how translation of non-English documents may have affected results. | Thank you for your comment. We have added a little additional information in the method section to address the creation of the dictionary and expanded the limitations to reflect the translation issue. The handling of ambiguous terms/false positives was already touched upon in the method section; we rephrased this a bit, but are happy to provide even more examples. |
| 4. Strengthening the policy implications  The discussion compellingly argues that superficial references to vulnerable groups risk reinforcing inequalities. Including concrete examples of what “equity-by-design” could look like in digital health strategies (e.g., explicit equity targets, digital literacy initiatives, accessibility standards) would enhance policy relevance. | Thank you for pointing this out (similar to reviewer 1). We have added a sentence in the discussion as well as suggestions for practice in the conclusion. |
| 5. Scope and generalizability  While the inclusion of 13 national strategies is reasonable, the discussion could more explicitly acknowledge regional diversity across Africa and clarify that findings reflect available and recent strategies rather than all national approaches. | Thank you for this comment. We have now explicitly mentioned this point in the limitations. |
| Minor Comments  - A light proofreading pass would address minor typographical and formatting issues.  - Some long sentences could be shortened for readability.  - Table 3 is informative but dense; simplifying it or moving some content to the appendix may improve clarity. | We proofread the document and hope that it is now free of any errors. We have also tried to shorten some longer sentence, as also suggested by the other reviewer. We also agree that table 3 is quite dense. We have decided to split the table according to the higher-order coding to make it less dense. We hope that this improves clarity. |
|  |  |

| **Decision Letter** | **Authors’ Response** |
| --- | --- |
| Thank you for submitting your manuscript to PLOS ONE. I have now completed a full assessment of your work, along with the detailed reports from our four reviewers. I commend you on a timely and methodologically innovative study that addresses a critical gap in digital health policy analysis within the African context. The application of computational text analysis to evaluate the inclusiveness of national strategies is a significant strength.  Overall Assessment:  Your manuscript presents a well-structured and conceptually sound inquiry into how African digital health strategies conceptualize and integrate the needs of vulnerable populations. The findings that these strategies prioritize systemic infrastructure while offering only superficial or program-specific attention to groups like women, children, and older adults are important, empirically supported, and have clear implications for health equity. The paper is well-situated within relevant literature and makes a valuable contribution to the fields of digital health, health policy, and equity studies.  Critical Synthesis of Reviewer Feedback:  All four reviewers recognized the manuscript's strengths and potential. Their feedback converges on several key areas requiring refinement to elevate the manuscript to the standard required for publication in PLOS ONE. The primary concerns are not foundational flaws but relate to scope justification, methodological transparency, interpretative nuance, and actionable policy discourse. | // |
| Major Revisions Required:  Strengthen Justification for Sample Scope and Selection:  The decision to limit the analysis to 13 post-2020 strategies is a significant delimitation. As raised by Reviewer 3, you must provide a more robust and explicit justification. Move beyond simply citing the WHO 2020 strategy. Discuss the trade-off: analyzing a contemporary, policy-coherent cohort versus achieving broader continental representativeness. Acknowledge this choice limits generalizability to “recent, formally articulated national strategies” rather than “African digital health policy” broadly. A sentence in the limitations should explicitly state that pre-2020 strategies and countries without formal published strategies are excluded, which may bias the findings toward more digitally advanced nations. | Thank you for this suggestion. As also suggested by the reviewers, we have strengthened the sample scope and justification by highlighting the focus on recent developments, adapting the methodology and extending the limitations (see also the responses to the other reviewers concerning this topic). |
| Clarify and Defend the Conceptualization of "Vulnerable Populations":  Reviewers 2, 3, and 4 correctly note that your operational definition (women, children, older adults) is narrow. This is a valid methodological choice, but it must be explicitly framed and defended. In the introduction or methods, add a paragraph justifying this focus. You could argue that these groups represent large, demographically definable categories where structural marginalization in health is well-documented, making them a logical starting point for a quantitative text analysis. Explicitly state that other crucial dimensions of vulnerability (disability, rurality, ethnicity, refugee status) are excluded from this analysis but represent vital areas for future qualitative or more granular computational research. This turns a limitation into a clarified scope. | Thank you for your suggestions. Based on your and the reviewers’ comments, we have extended our explanation on the choice of focusing solely on women, elderly and youth as well as extending our limitation section. |
| Enhance Methodological Transparency and Reproducibility (PLOS ONE Mandate):  Reviewer 3's request for data and code availability is non-negotiable for computational studies. You must state in the Data Availability Statement that the following have been deposited in a public repository (e.g., GitHub, Zenodo, OSF):  The anonymized/pre-processed corpus of strategy texts.  The final dictionary/lists of terms used for the frequency analysis.  The analysis scripts (Python/R code) used for LDA topic modeling and dictionary-based counting, including all parameter settings. | We have published the supplementary material under this link: <https://doi.org/10.6084/m9.figshare.31169590>  We have included the link in the paper, referring to “supplementary material available online” in the methods section. |
| Furthermore, in the Methods, briefly describe the development and validation process for your dictionaries. Were terms generated from literature? Was there an iterative review process? How were synonyms or multi-word phrases handled? | Thank you for pointing out the lack of necessary descriptions. The included dictionary terms are based on own prior research and relevant terms used by the WHO to describe the included vulnerable populations. Multi-word phrases were included in the dictionary as well and counted as “one” term (e.g. “60 and above” would count as one mention of the elderly). The counted terms were manually reviewed to ensure the accuracy of the dictionary results. We tried to make this clearer in the methods section. We have also expanded the limitations section to more explicitly address the constraints of the dictionary-based approach, particularly the risk that predefined dictionaries may not capture all relevant terms. |
| Beyond Identification to Mechanism: The discussion effectively identifies gaps but should go further. Why might policymakers default to superficial mentions? Link this to broader governance challenges, resource constraints, or the technical-infrastucture focus of digital health discourse. This elevates the analysis from descriptive to explanatory. | Thank you for this input. We have extended our discussion and included specific examples how language in digital health strategies can be tailored to specifically reflect the needs of each included vulnerable populations. |
| From "What's Missing" to "How to Include": Reviewers 1 and 4 request more actionable guidance. Strengthen the conclusion by moving from “strategies should consider equity” to "strategies could operationalize equity by, for example, mandating accessibility standards in procurement, including equity indicators in M&E frameworks, or budgeting for targeted digital literacy programs for older adults.” Provide 2-3 specific, concrete examples of what “equity-by-design” language would look like in a policy document. |  |
| Acknowledge the Limits of Topic Modeling: As Reviewer 4 notes, the absence of a “vulnerable populations” topic does not equate to total neglect. Add a sentence in the results or discussion acknowledging that mentions may be embedded within other topics, but the fact they do not coalesce into a dedicated thematic priority is itself a significant finding regarding strategic focus. | We have included this in the discussion. |
| Source Clarification: Resolve the query from Reviewer 3 regarding quotes about healthcare worker burdens (Lines 233-234, 237-240). If these are from the strategy documents, cite the specific document. If they are from secondary sources referenced within the strategies, clarify this. If they are external data inadvertently included, they should be removed or explicitly sourced. | This was a misunderstanding. We responded to the respective comment and tried to clarify this more in the manuscript. |
| Terminology: Standardize the use of “vulnerable populations,” “marginalized groups," etc., early in the manuscript for consistency. | Done. |
| Table and Text Editing: Fix the truncated text in Table 3. Heed Reviewer 1's advice to break down overly long, dense sentences to improve readability. Conduct a thorough proofread. | Done. |

| **Journal Requirements** | **Authors’ Response** |
| --- | --- |
| We noted in your submission details that a portion of your manuscript may have been presented or published elsewhere. “The first and last author (Kimsey Zajac and Lutz Kolbe) authored a similar paper, which was published at the European Conference on Information Systems 2024; while there are similarities across the data and method, the focus of this manuscript is different. The paper is uploaded with the submission.” Please clarify whether this [conference proceeding or publication] was peer-reviewed and formally published. If this work was previously peer-reviewed and published, in the cover letter please provide the reason that this work does not constitute dual publication and should be included in the current manuscript. | We extended our reasoning for an updated manuscript in the new letter to the editor. The main points are also briefly summarized here:  - Scope: Conference paper: Global analysis across 4 income groups; PLOS One paper: Focused analysis on African strategies for deeper contextual insight.  - Temporal Focus: Conference paper: Broad timeframe (mid-2000s–2023); PLOS One paper: Strictly post-2020 strategies to align with the WHO’s 2020-2025 Global Strategy.  - Key Findings: Conference paper reveals income-based differences in strategic priorities and inclusivity; PLOS One identifies a gap between acknowledging vulnerability and implementing concrete equity measures in African policies. |
| Please note that your Data Availability Statement is currently missing [the repository name and/or the DOI/accession number of each dataset OR a direct link to access each database]. If your manuscript is accepted for publication, you will be asked to provide these details on a very short timeline. We therefore suggest that you provide this information now, though we will not hold up the peer review process if you are unable. | The DOI-link for the data and analysis scripts is now included in the paper. |
| Please ensure that you refer to Figure 2 in your text as, if accepted, production will need this reference to link the reader to the figure. | This was a typo, the reference in the manuscript was meant to refer to Figure 1. We have adapted this. |
| If the reviewer comments include a recommendation to cite specific previously published works, please review and evaluate these publications to determine whether they are relevant and should be cited. There is no requirement to cite these works unless the editor has indicated otherwise. | // |
