# Supplementary material for: A computational text analysis of recent African digital health strategies and their attention to vulnerable populations
Source: PLoS One. 2026 May 19;21(5):e0348593. doi: 10.1371/journal.pone.0348593 (PMC13186356; doi:10.1371/journal.pone.0348593)
Supplement: Attachment — Submitted filename: Response_to_Reviewers_auresp_3.docx [file pone.0348593.s007.docx]

Dear Dr. Raimi, dear editorial team,

Thank you for your valuable comments on our manuscript and to the Academic Editor for facilitating the revision process.

We have made the necessary changes to our manuscript, and believe that your feedback helped improve our submission immensely. Please find our point-by-point responses to the reviewer comments and the required changes according to the decision letter in the response tables below.

Sincerely,

Kimsey Zajac, Louisa Peters, Lutz Kolbe

| **Decision Letter** | **Authors’ Response** |
| --- | --- |
| Dear Authors,  Thank you for submitting your revised manuscript entitled “A computational text analysis of recent African digital health strategies and their attention to vulnerable populations.” I have carefully reviewed your revised submission, the detailed point-by-point response to the four external reviewers, and your responses to the specific requirements outlined in my previous decision letter (Major Revision). I have also examined the changes made to the manuscript, including the new methodological clarifications, the expanded limitations section, the added policy recommendations, and the public deposition of your analysis scripts and preprocessed text corpus.  I am pleased to state that you have addressed the vast majority of the concerns raised during the peer review process in a thorough, professional, and responsive manner. The manuscript is now substantially stronger in its methodological transparency, interpretive nuance, and policy relevance. The decision to focus on post-2020 strategies is now better justified, the operational definition of “vulnerable populations” is more clearly defended, and the discussion has been deepened with actionable recommendations for equity-by-design. However, despite these significant improvements, three minor but necessary issues remain, none requiring new analysis or conceptual revision, but all requiring correction before final acceptance. These relate to formatting, a missing figure reference, and a minor inconsistency in terminology.  Below, I provide my final decision, a synthesis of what has been done well, the outstanding minor issues, and specific actionable requirements. | Thank you for your support! |
| I. Summary of What Has Been Done Well  1. Sample justification strengthened: You now explicitly acknowledge the trade-off between contemporary policy coherence (post-2020, aligned with WHO Global Strategy) and broader continental representativeness, and you have added a limitation stating that findings apply to “recent, formally articulated national strategies” rather than African digital health policy broadly.  2. Vulnerable populations framing clarified: You have added a clear justification for focusing on women, children/youth, and older adults as large, demographically definable groups with well-documented structural marginalization, while explicitly stating that other dimensions (disability, rurality, ethnicity, refugee status) are excluded and represent vital areas for future research.  3. Methodological transparency achieved: You have deposited the preprocessed text corpus, dictionary terms, and Python analysis scripts in a public repository (Figshare, DOI: 10.6084/m9.figshare.31169590), fulfilling PLOS One’s reproducibility mandate.  4. Discussion deepened with mechanism and action: You now explain why policymakers may default to superficial mentions (resource constraints, donor priorities, technical framing), and you provide concrete examples of equity-by-design language (e.g., accessibility standards, equity indicators in M&E frameworks, targeted digital literacy programs for older adults).  5. Topic modeling limits acknowledged: You now explicitly note that the absence of a “vulnerable populations” topic does not equate to total neglect but indicates insufficient salience to emerge as a standalone theme – a finding in itself.  6. Quotes clarified and cited: You have resolved the ambiguity regarding quotes about healthcare worker burdens by confirming they are from the strategy documents and have cited the specific documents (Zambia, Malawi, etc.).  7. Table 3 restructured: The dense table has been split according to higher-order coding, improving readability.  8. Long sentences shortened and repetition reduced: The manuscript now reads more smoothly. | // |
| II. Outstanding Minor Issues Requiring Correction Before Acceptance  Issue 1: Missing Figure 1 Reference and Missing Figure 2  Problem:  In the revised manuscript, you refer to “Figure 1” in the text (e.g., on page 17, line 224: “The model with nine topics was found to be optimal (see Figure 1)”). However, the manuscript file contains no visible Figure 1 – only a blank placeholder or a corrupted image appears on page 52. Additionally, the inter-topic distance map (which appears to be intended as Figure 1) is not clearly labeled. On page 52, there is a small, low-resolution image labeled “3 PC2 5 PC1 8 9 7 4 6 Marginal topic distribution 2 1 2% 5% 10%” – this is not interpretable as a proper figure. Furthermore, Figure 2 is referenced in the text (page 19, line 255: “The results from the inter-topical distance map (see Fig 1) indicate…” but there is no reference to Figure 2 anywhere. The dictionary-based results table is labeled Table 7, not a figure.  Required Action:  • Provide a clear, high-resolution Figure 1 (inter-topic distance map) with a proper title, axis labels, and legend. Ensure it is embedded correctly in the manuscript file.  • If the inter-topic distance map is the only figure, renumber it as Figure 1 and remove any reference to Figure 2. Alternatively, if you intended a second figure (e.g., a bar chart of topic proportions), please provide it and reference it in the text.  • Ensure that all figures are visible, legible, and properly captioned. | Figure 1 is provided separately from the manuscript as per PLOS One Journal Requirements. A high-resolution figure of the inter-topic distance map is uploaded in the system (we have re-uploaded Figure 1). The figure on page 52 is Figure 1.  We do not have a Figure 2 and we double-checked: no Fig.2 is referenced in the manuscript. This was an issue with our first submission, but it does not appear to be a problem in this version. |
| Issue 2: Inconsistent Terminology for “Vulnerable Populations” vs. “Structurally Marginalized Groups”  Problem:  The title, abstract, and introduction use “vulnerable populations” consistently, but the discussion and conclusion occasionally use “structurally marginalized groups” or “disadvantaged groups” interchangeably. While not a fatal flaw, this inconsistency may confuse readers. The Academic Editor’s decision letter explicitly requested: “Standardize the use of ‘vulnerable populations,’ ‘marginalized groups,’ etc., early in the manuscript for consistency.”  Required Action:  • Choose one primary term – “vulnerable populations” is appropriate – and use it consistently throughout the manuscript. Replace instances of “structurally marginalized populations,” “disadvantaged groups,” etc., with “vulnerable populations” unless a specific distinction is intended. If a distinction is intended (e.g., referring to a subset), define it clearly. | Thank you for your comment. We only identified one instance where we referred to “vulnerable populations” as “disadvantaged groups” (line 65) and none were we mentioned “marginalized groups” or similar. We rectified the issue and changed the terminology in line 65. |
| Issue 3: Minor Formatting and Typographical Errors  Problem:  • Page 73, line 389: A stray formatting character appears (“limited stakeholders’ involvement during planning, design, and implementation”). This appears to be a copy-paste artifact. | We have changed some formatting in the quote. |
| • Page 76, line 444: The quote from Malawi is repeated twice in close succession (lines 444–447 and 448–451) – appears to be a duplication error. | The text before the quote explained the content of the quote, which likely added to the appearance of this quote being inserted twice. We paraphrased the text so that the appearance of duplication is limited. |
| • Table 7 (page 80) includes a row “Strategies not mentioning group,” but the formatting is inconsistent (some countries listed with commas, others with “and”). Standardize. | This does not seem to be an issue; we can not find an “and” in the table. |
| Required Action:  • Remove the stray formatting character on page 73.  • Delete the duplicate quote on page 76 (lines 448-451).  • Standardize the “Strategies not mentioning group” row in Table 7 (e.g., use commas consistently, no “and” within the list). | Done. |
| III. Decision Justification  This manuscript has undergone two full rounds of peer review with four external reviewers and a detailed Major Revision decision from the Academic Editor. The authors have responded comprehensively and professionally, addressing virtually all substantive concerns. The manuscript now makes a clear, empirically supported, and policy-relevant contribution to the literature on digital health equity in Africa.  The remaining issues are minor and purely editorial: a missing/corrupted figure, inconsistent terminology, and a few formatting errors. These do not affect the scientific validity or interpretability of the findings but must be corrected for publication.  Therefore, I recommend Accept with Minor Revision. | // |
| IV. Specific Action Items for Final Revision (Mandatory)  1. Provide a clear, high-resolution Figure 1 (inter-topic distance map) with proper title, axis labels, and legend. Ensure it is embedded correctly and visible.  2. Resolve figure numbering: If only one figure exists, renumber as Figure 1 and remove any references to Figure 2. If a second figure is intended, provide it and reference it.  3. Standardize terminology: Use “vulnerable populations” consistently throughout the manuscript. Replace “structurally marginalized groups,” “disadvantaged groups,” and similar variants unless a specific distinction is intended and defined.  4. Remove formatting artifact on page 73 (stray character before “limited stakeholders’ involvement”).  5. Delete duplicate quote on page 76 (lines 448–451).  6. Standardize Table 7 formatting – use consistent punctuation in the “Strategies not mentioning group” row. | 1. Figure is provided separately according to PLOS One rules and regulations. We uploaded a new version to the system.  2. No reference to figure 2 in this manuscript version.  3. Done.  4. We have adapted the formatting a bit. We did not identify a stray character. We think that there might have been the appearance of a stray character due to the system’s compilation of the manuscript. If this comment refers to the “[…]”, we have included the brackets to show that we have excluded parts of the original quote, so we chose to keep it to maintain transparency.  5. Fixed.  6. Non-issue (see above). |
| V. Final Editorial Comment  This study makes an important and timely contribution to the field of digital health policy analysis. The finding that recent African digital health strategies prioritize systemic infrastructure while offering only superficial attention to vulnerable populations – particularly older adults – is both concerning and actionable. Your use of computational text analysis (LDA + dictionary-based methods) is methodologically sound and serves as a model for similar policy evaluations in other regions. I commend you for your responsiveness to reviewer feedback, particularly your willingness to deposit your code and data publicly, to clarify your conceptual framing, and to provide concrete policy recommendations. These actions exemplify the transparency and practical impact that PLOS One seeks to promote. Upon receipt of the corrected figure, standardized terminology, and minor formatting fixes, I will recommend final acceptance without further peer review. | Thank you for valuable comments and your support! |
